# Oral Neutrophils Characterized: Chemotactic, Phagocytic, and Neutrophil Extracellular Trap (NET) Formation Properties

**DOI:** 10.3389/fimmu.2019.00635

**Published:** 2019-03-29

**Authors:** Carolyn G. J. Moonen, Josefine Hirschfeld, Lili Cheng, Iain L. C. Chapple, Bruno G. Loos, Elena A. Nicu

**Affiliations:** ^1^Department of Periodontology, Academic Centre for Dentistry Amsterdam (ACTA), University of Amsterdam and Vrije Universiteit Amsterdam, Amsterdam, Netherlands; ^2^Periodontal Research Group, Birmingham Dental School and Hospital, The University of Birmingham and Birmingham Community Health NHS Trust, Birmingham, United Kingdom; ^3^CMI Dr. Opris M.I., Sibiu, Romania

**Keywords:** polymorphonuclear leukocytes, migration, phagocytosis, PMN, chemotaxis

## Abstract

Maintenance of oral health is in part managed by the immune-surveillance and antimicrobial functions of polymorphonuclear leukocytes (PMNs), which migrate from the circulatory system through the oral mucosal tissues as oral PMNs (oPMNs). In any microorganism-rich ecosystem, such as the oral cavity, PMNs migrate toward various exogenous chemoattractants, phagocytose bacteria, and produce neutrophil extracellular traps (NETs) to immobilize and eliminate pathogens. PMNs obtained from the circulation through venipuncture (hereafter called cPMNs) have been widely studied using various functional assays. We aimed to study the potential of oPMNs in maintaining oral health and therefore compared their chemotactic and antimicrobial functions with cPMNs. To establish chemotactic, phagocytic, and NET forming capacities, oPMNs and cPMNs were isolated from healthy subjects without obvious oral inflammation. Directional chemotaxis toward the chemoattractant fMLP was analyzed using an Insall chamber and video microscopy. fMLP expression was assessed by flow cytometry. Phagocytosis was analyzed by flow cytometry, following PMN incubation with heat-inactivated FITC-labeled micro-organisms. Furthermore, agar plate-based killing assays were performed with *Escherichia coli* (*Ec*). NET formation by oPMNs and cPMNs was quantified fluorimetrically using SYTOX™ Green, following stimulation with either PMA or RPMI medium (unstimulated control). In contrast to cPMNs, the chemotactic responses of oPMNs to fMLP did not differ from controls (mean velocity ± SEM of cPMNs: 0.79 ± 0.24; of oPMNs; 0.10 ± 0.07 micrometer/min). The impaired directional movement toward fMLP by oPMNs was explained by significantly lower fMLP receptor expression. Increased adhesion and internalization of various micro-organisms by oPMNs was observed. oPMNs formed 13 times more NETs than stimulated cPMNs, in both unstimulated and stimulated conditions. Compared to cPMNs, oPMNs showed a limited ability for intracellular killing of *Ec*. In conclusion, oPMNs showed exhausted capacity for efficient chemotaxis toward fMLP which may be the result of migration through the oral tissues into the oral cavity, being a highly “hostile” ecosystem. Overall, oPMNs' behavior is consistent with hyperactivity and frustrated killing. Nevertheless, oPMNs most likely contribute to maintaining a balanced oral ecosystem, as their ability to internalize microbes in conjunction with their abundant NET production remains after entering the oral cavity.

## Introduction

Polymorphonuclear leukocytes (PMNs) are terminally differentiated innate immune cells that descend from hematopoietic stem cells in the bone marrow and transit through the peripheral blood circulation as circulatory PMNs (cPMNs) (1, 2). Traditionally, cPMNs exit the vasculature and migrate to sites of tissue damage, inflammation, and infection to perform various protective and antimicrobial functions contributing to the neutralization and elimination of pathogens and damaged cells (3).

The oral cavity typically harbors over 700 different species of colonizing bacteria evidently priming and activating the PMNs, which originate from a nearly sterile blood circulation (4). In a healthy state, approximately 30,000 oral PMNs (oPMNs) per minute arrive through the gingival crevicular fluid (GCF) which flows into the oral cavity from the periodontal sulcus (5). In the gingival crevice, oPMNs form a wall between the epithelium and the dental biofilm to protect the periodontal tissue and to maintain periodontal tissue homeostasis (6–8). Moreover, oPMNs migrate from all oral mucosal tissues (9). It has been suggested that in the oral cavity, PMNs carry out a unique immune surveillance function and symbiotically interact with the commensal oral microflora in order to maintain homeostasis and oral health (10). In cases of chronic inflammation, such as in periodontitis, a multifactorial chronic inflammatory disease of the periodontium leading to alveolar bone loss (11), an increased influx of oPMNs with a hyperactive phenotype extravasate into the oral cavity (12).

PMNs recognize, immobilize, internalize, and kill extracellular pathogens both intra- and extracellularly (13, 14). PMN recruitment and migration is mediated by various endogenous and exogenous chemoattractants such as interleukin-8 (IL-8), bacteria-derived lipopolysaccharides (LPS), and N-formyl-methionyl-leucyl-phenylalanine (fMLP). These chemoattractants initiate signal transduction events, leading to a multitude of cellular processes including diapedesis, chemotaxis, and migration of PMNs (15, 16). Upon contact, PMNs adhere to and engulf bacteria, a process known as phagocytosis (17). Impaired phagocytic capacities of PMNs lead to the accumulation of bacteria, delay of bacterial clearance and disturbance of oral microbial homeostasis (18). Pathogen destruction is for a large part accomplished through reactive oxygen species (ROS) generation by activated PMNs. Another antimicrobial strategy of PMNs is the formation of neutrophil extracellular traps (NETs) released by highly activated neutrophils (19–21). NETs are formed after nuclear membrane and granule disintegration. Subsequently, nuclear material and contents of the granules mix, and NETs are released after cell membrane disruption, followed by cell death. This has been termed suicidal NETosis (21, 22). It has been reported that PMNs can also release NETs without cell membrane rupture. Since these PMNs remain viable, it is called vital NETosis (23). NETs consist of a core DNA element, decondensed nuclear chromatin combined with various DNA-bound antimicrobial proteins and peptides (24). The putative role of NETs is to entrap pathogens and they are induced by diverse stimuli such as microbes, host factors, microbial products, and immune complexes which bind to the receptors on the surface of PMNs (25).

Hitherto, blood-derived PMNs (cPMNs) have been extensively studied in relation to the pathogenesis of oral inflammation, and to periodontitis in particular (26, 27). Several studies show that altered PMN activity, including impaired chemotaxis (16), phagocytosis (28), and NET formation (25, 29, 30), is associated with the disruption of tissue homeostasis and disease. However, few studies are available about the functional antimicrobial characteristics of oPMNs. Glogauer et al. found elevated numbers of oPMNs with a hyperactive phenotype in periodontitis patients reflecting the severity of periodontal disease and treatment response (31–33). It was confirmed by our group that oPMNs are hyperactive cells in the oral ecosystem by demonstrating a significant increase in ROS production (7). However, oPMNs' characteristics such as the chemotaxis, phagocytosis, and NET formation have yet to be fully elucidated. In this study, we aimed to compare oPMNs and cPMNs, in terms of their chemotactic, phagocytic, and NET forming behavior.

## Materials and Methods

### Human Subjects and Experimental Setup

Donors (*n* = 9) for the chemotaxis, NET formation (*n* = 9), and killing (*n* = 4) experiments were recruited at the Birmingham Dental School and Hospital (United Kingdom). For the phagocytosis and fMLP expression experiments, donors (*n* = 3) were recruited at the Academic Center for Dentistry Amsterdam (ACTA, The Netherlands). The study was approved by the Birmingham ethical committee (14/SW/1148) and the Medical Ethical Committee of the Amsterdam University Medical Center, The Netherlands (2012-210#B2012406). All donors were systemically and periodontally healthy. Informed and written consent was obtained from all individuals prior to inclusion. Venous blood samples and oral rinses from each donor were collected. cPMNs and oPMNs were isolated and experiments were performed on the same day without delay.

### cPMN Isolation

Venous blood from healthy individuals was obtained in lithium heparin tubes (Vacuette® Heparin tubes, Greiner Bio-One, Alphen a/d Rijn, The Netherlands). Blood was diluted 1:1 in 1% PBS citrate (pH 7.4). Subsequently, 25 mL of the diluted blood was carefully layered on 15 mL Lymphoprep (Axis-shield Po CAS, Oslo, Norway) and centrifuged for 30 min at 800 RCF without brake. The supernatant above the red cell layer was removed, after which remaining erythrocytes were lysed in cold lysis buffer (NH_4_Cl [1.5 M], NaHCO_3_ [100 mM], disodium EDTA [1 mM] H_2_O, all Sigma-Aldrich, Merck, Darmstadt, Germany, 10 x diluted in sterile MQ water). The cPMN pellet was washed twice in cold PBS (Gibco, Thermo Fischer Scientific, Paisley, Scotland, UK) immediately after erythrocyte lysis and recovered in culture medium (phenol-red free, Roswell Park Memorial Institute [RPMI] 1640, Gibco). cPMN counts and viability were determined with the Muse® Count & Viability Assay Kit using the Muse™ cell analyzer and its associated Count & Viability Software Module (Millipore, Merck, Burlington, Massachusetts, USA) according to the manufacturer's instructions. Additionally, cell viability was microscopically confirmed with propidium iodide (PI, Life Technologies by Thermo Fischer) staining (500 ng/mL, 5 min at RT). Images were acquired using a 20 x objective (Leica Microsystems, DM2000, Zeiss, Germany). Leica software (Version 4.2) was used for image acquisition and processing. Representative viability micrographs and data are presented in Supplementary Figure 1.

### oPMN Isolation

Oral rinses were collected and isolated according to previously described protocols (7, 9). Briefly, 4 oral rinses of 30 s with 10 mL of sterile 0.9% sodium chloride solution (Versylene®, Fresenius Kabi, Sèvres, France) with intermission periods of 4 min were collected. Oral rinses were centrifuged at 500 RCF, after which the pellet was suspended in 10 mL PBS. Next, the oral samples were subsequently filtered through 70.0, 40.0, 31.5, and 10.0 micrometer (μm) nylon meshes (Vlint, Nedfilter, Almere, The Netherlands) to exclude epithelial cells and cell debris. The filtrated fraction was centrifuged (500 RCF for 10 min at 4°C), washed in cold PBS and suspended in culture medium. oPMNs were counted and checked for viability with the Muse® Count & Viability Assay Kit using the Muse™ cell analyzer and its associated Count & Viability Software Module according to the manufacturer's instructions. Additionally, cell viability was microscopically confirmed with a PI staining (500 ng/mL, 5 min at RT). Images were acquired using a 20 x objective (Leica Microsystems). Leica software (Version 4.2) was used for image acquisition and processing. Representative viability micrographs and data are presented in Supplementary Figure 1.

### Chemotaxis Assay

Chemotaxis experiments were performed as described previously by Roberts et al. (16). Briefly, the chemotactic movements of oPMNs and cPMNs (*n* = 9) were investigated over time in Insall chambers (34). Coverslips (Borosilicate glass, thickness number 1.5, VWR International, Radnor, PA, USA) were coated with 10% bovine serum albumin (BSA) in PBS for 1 min after washes with hydrochloric acid (0.2 M in H_2_O) and sterile water. Subsequently, 3 × 10^5^ PMNs/mL were added onto the glass slide and allowed to attach for 20 min. Chemoattractant (15 nM fMLP) or control (PBS) was added to the chamber in which the cells were observed with a Zeiss Primovert video microscope (Carl Zeiss Imaging, Thornwood, NY, USA). Images were captured at a 20 x magnification for 20 min with a time interval of 30 s using a Q Imaging Retiga 2000R camera (Qimaging, Surrey, BC, Canada).

### Time-Lapse Image Analysis

Time-lapse images, generated by video microscopy, were processed using Q pro-imaging software (Qimaging) and analyzed using Fiji (35) with the MtrackJ plugin (36), where 15 random cells were tracked per condition. Generated numerical data was used to calculate cell speed, cell velocity and chemotactic index (CI). XY coordinates of the cells were analyzed with circular statistics using MATLAB (Version 2017b, The Mathworks, Natick, MA, USA). Directional data is presented in one spider plot (created using Microsoft Excel, Microsoft office 2010) and two separate circular diagrams: vector plots and rose plots (created using MATLAB). Vector plots indicate the distribution per proportion of cells in each of the 12 segments around the circle. The direction of the longest segment shows the direction with the greatest frequency. The distribution of the final angle of all cells with a vector line showing the median angle (red vector), including the interquartile range (interrupting blue vectors) is presented in rose plots. Additionally, cPMN's and oPMN's chemotactic index, speed and velocity were calculated and are presented in Tukey box and whisker plots. The chemotactic index was calculated as a change in the angle of a cell along the y-axis according to the cosine plot (34). The speed and velocity was the average speed of cPMNs and oPMNs in any direction or in its most prominent direction over the time course, respectively.

### fMLP Receptor Expression

Following isolation, cPMNs and oPMNs were stained (30 min, 4°C in the dark) with APC-conjugated anti-human CD16 (clone 3G8, BD Biosciences, Piscataway, NJ, USA) and FITC-conjugated anti-human CD66b (clone G10F5, BD Biosciences). To investigate fMLP receptor expression, cells were stained with FITC-conjugated anti-human fMLP receptor (clone REA169, Miltenyi Biotec, Bergisch Gladbach, Germany) or FITC-labeled anti-human IgG1κ (clone MOPC-21, BD Biosciences) as an isotype control. After incubation, flow cytometric acquisition and analysis were performed on a BD FACSverse™ flow cytometer (BD Biosciences) with a medium flow rate (63 μL/min). Flow cytometry data were analyzed using associated FACSuite software (Version 1.0.5, BD Biosciences).

The gating strategy employed is shown in Supplementary Figure 2. Briefly, the live population of cPMNs (Supplementary Figure 2A) and oPMNs (Supplementary Figure 2B) was determined based on forward- (FSC) and sideward scatter (SSC), which, respectively represented the distribution of cells in the light scatter based on size and intracellular composition. cPMNs and oPMNs were tested for CD16 (cPMNs: 99.7%, oPMNs: 96.2%) and CD66b (cPMNs: 99.8%, oPMNs; 80.4%) as neutrophil markers (37), where CD16-negative and CD66b-negative gates were determined using unstained samples. Finally, in the CD16-positive population, fMLP receptor expression was assessed on cPMNs and oPMNs. fMLP receptor negative gates were set in control conditions and stained with the corresponding isotype IgG1κ.

### Strains and Growth Conditions

Micro-organisms were commercially obtained (DSMZ, Braunschweig, Germany). For this study, 6 oral bacteria and 1 yeast strain were selected; *Aggregatibacter actinomycetemcomitans* (*Aa*, strain Y4), *Porphyromonas gingivalis* (*Pg*, strain W83), *Fusobacterium nucleatum (Fn*, strain ATCC10953*), Tannerella forsythia (Tf*, strain ATCC 43037*), Streptococcus mutans* (*Sm*, strain UA159)*, Streptococcus sanguinis* (*Ss*, strain HG1470*)*, and *Candida albicans (Ca*, strain SC5314*)*. Furthermore, the non-oral bacteria *Escherichia coli* (*Ec*, strain DSM 18039*)* was included.

*Aa, Pg*, and *Fn* were grown anaerobically (80% nitrogen, 10% carbon dioxide, and 10% hydrogen) in enriched brain-heart infusion (enriched BHI; 5 μg/mL hemin, 1 μg/mL menadione, Sigma-Aldrich Chemie B.V., Zwijndrecht, Netherlands). *Ca* cultures were grown aerobically in yeast-peptone-glucose (BBL) in an orbital shaker (120 RPM, 30°C, overnight). *Tf* was anaerobically grown in enriched BHI supplemented with 5% fetal calf serum, 1 gram/L L-cysteine, and 15 mg/L N-acetylmuramic acid. *Sm* was grown aerobically at 37°C in BHI. *Ss* was grown aerobically in BHI. *Ec* was grown aerobically at 37°C in Luria-Bertani broth.

Bacterial and yeast suspensions were isolated from broth cultures by centrifugation, washed twice in sterile PBS and diluted to an optical density of 1 at 600 nm in 4-(2-hydroxyethyl)-1-piperazineethanesulfonic acid (HEPES) buffer. Microbes were heat-inactivated at 60°C for 1 h and stored in aliquots at −20°C for further analyses.

### Adhesion and Internalization Assays

Micro-organisms were extrinsically labeled for 1 h with 100 μg/mL fluorescein isothiocyanate (FITC, Sigma Aldrich) and washed in phosphate buffer (PB, 0.1 M, pH 7.2) to remove remaining unlabeled FITC molecules. Labeling efficiency (>70%) was defined by flow cytometry before tests.

Adhesion and internalization by cPMNs and oPMNs were flow cytometrically analyzed. Both adhesion and internalization assays were performed with un-opsonized heat-inactivated microbes. Immediately after isolation, PMNs were recovered in HEPES buffer (pH 7.4), supplemented with 1 mM calcium chloride, 0.5% BSA, and 1 mg/mL glucose (all Sigma-Aldrich). FITC-conjugated anti-human CD66b (clone G10F5, BD Biosciences) was used to check for neutrophil purity (37). PMNs were stained with APC-conjugated anti-human CD16 (Clone 3G8, BD Biosciences) for 30 min in the dark. Cells were incubated with heat-inactivated FITC labeled microbes at a previously established optimal PMN/microbe ratio of 1:3 for 30 min at 4 or 37°C for adhesion and internalization, respectively. Internalization was distinguished from adhesion using the membrane-impermeable dye trypan blue (38). Accordingly, trypan blue (20 μg/mL, Sigma Aldrich) was added to quench the fluorescence of adherent FITC-labeled micro-organisms before the assessment of internalization. After 30 min of incubation, cells were fixed in 1% paraformaldehyde (PFA) in PBS. Flow cytometric acquisition and analysis was performed on a BD FACSverse™ flow cytometer (BD Biosciences) with a medium flow rate (63 μL/min) where at least 10,000 cells were analyzed per sample. Flow cytometry data were analyzed using associated FACSuite software (BD Biosciences).

The gating strategy employed for adhesion and internalization assays is presented in Supplementary Figure 3. Briefly, the live population of cPMNs (Supplementary Figure 3A) and oPMNs (Supplementary Figure 3B) was determined based on forward- (FSC) and sideward scatter (SSC), which, respectively represented the distribution of cells in the light scatter based on size and intracellular composition. Subsequently, cPMNs and oPMNs were tested for CD16 (cPMNs: 99.8%, oPMNs: 96.8%) and CD66b (cPMNs: 99.8%, oPMNs; 80.4%) as neutrophil markers (37), where CD16-negative and CD66b-negative gates were determined using unstained samples. Finally, adhesion and internalization of FITC-labeled bacteria by PMNs was determined based on the percentage of FITC+CD16+ population. Accordingly, this population illustrated the percentage of FITC+ events (i.e., FITC-labeled microbes) detected in the CD16+ (i.e., PMN) population.

### Killing Assay

In order to assess the killing capacity of cPMNs and oPMNs, phagocytosis assays were performed with live *Ec (*strain W3110, ATCC 27325). As oPMN samples contained numerous oral bacteria, we pre-incubated isolated oPMNs with an antibiotic and antifungal cocktail (final concentration of 2.5 μg/mL Amoxicillin, 25 μg/mL Tetracycline, 25 μg/mL Metronidazole, 2.5 μg/mL Fungizone, all Sigma-Aldrich). After 15 min of incubation at RT, oPMNs were washed in 20 mL PBS. After centrifugation (10 min at 500 RCF), the supernatant was stored to test for the possible presence of antibiotics in the oPMN samples which would impact *Ec* survival.

*Ec* was grown on BHI agar. A single colony was inoculated into BHI broth and grown overnight at 37°C on a shaker. The following day, 20 μL of the bacterial suspension was inoculated into 20 mL of fresh BHI broth and *Ec* was grown at 37°C on a shaker until its late log phase (OD600 = 0.9–1.0). Next, *Ec* was added to 250,000 PMNs (PMN/bacteria ratio of 1:3) and incubated at 37°C in a shaking water bath. After 30 min of incubation, the cell-bacteria suspension was diluted 1000 x in PBS and plated (100 μL per plate) onto BHI agar plates in triplicate and grown overnight at 37°C, 5% CO_2_. Additionally, cell counts and the viability of cPMNs and oPMNs was routinely assessed with trypan blue light microscopy which resulted in viabilities of 93.35% (cPMNs) and 65.18% (oPMNs) after incubation with bacteria (mean percentages, *n* = 4). Finally, colony forming units (CFU) were counted.

Several controls were included for killing experiments. As a first control representing 0% killing, *Ec* was incubated and plated alone. As a second control condition, oPMNs were plated in order to determine the colonies originating from oral bacteria, which had survived incubation with antibiotics. This resulted in up to 5 CFU per plate, demonstrating that antibiotics were sufficient in killing oral bacteria in oPMN samples. Lastly, *Ec* was incubated with supernatants from the oPMN washing step after incubation with antibiotics, in order to investigate whether traces of antibiotics in the PMN pellets would affect bacterial killing of *Ec*. However, no differences were observed between *Ec* alone and *Ec* incubated with supernatants, proving that, even if present, any traces of antibiotics in the oPMN samples had negligible concentrations as they did not affect the survival of *Ec*.

### Visualization of NET Formation by cPMNs and oPMNs

cPMNs and oPMNs (1 x 10^6^ cells in culture medium) were added to a transparent 48-wells plate (Greiner Bio-One, Alphen a/d Rijn, The Netherlands), previously coated with filter sterilized 1% BSA. After 30 min of baseline incubation (37°C, 5% CO_2_), selected wells were stimulated with either 75 nM phorbol 12-myristate-13-acetate (PMA, Sigma Aldrich, Merck) or culture medium as control and incubated for 3 h in a humidified atmosphere of 5% CO_2_ in air at 37°C. Post-incubation, 50 nM extracellular nucleic acid dye SYTOX™ green (Invitrogen by Thermo Fisher Scientific, California, USA) was added to each well for the visualization of NETs using a fluorescence microscope (Leica Microsystems). The morphology of PMNs forming NETs was characterized by the release of extracellular web-like DNA strands. Images were acquired using 20 x and 40 x objectives (Leica Microsystems). Leica software (Version 4.2) was used for image acquisition and processing.

### Quantification of NET Formation

cPMNs and oPMNs (1 × 10^5^ cells in culture medium) were added to white, flat-bottom, non-treated, 96-wells plates (Greiner Bio-one) previously coated with filter-sterilized 1% BSA in PBS. After 30 min of baseline incubation at 37°C, selected wells were stimulated with 75 nM PMA for NET formation. Additionally, PMNs were incubated with culture medium as non-stimulated control. After 3 h of incubation at 37°C, 5% CO_2_, 1 U/mL micrococcal nuclease (MNase, Invitrogen by Thermo Fischer Scientific) was added and incubated for 15 min at 37°C to digest any PMN bound NET DNA. Cells and debris were pelleted (1800 RCF, 10 min), after which the supernatant was transferred to a black non-treated 96-wells plate containing 50 nM of the extracellular DNA nucleic acid dye SYTOX™ green (Invitrogen by Thermo Fischer Scientific) for the fluorimetric quantification of free NET-DNA fragments. Fluorescence was read in arbitrary fluorescence units (AFU) using a fluorospectrophotometer (Twinkle LB 970, Berthold Technologies, Oak Ridge, TN, USA), with an excitation of 485 nm and emission of 525 nm, at 37°C. All samples were tested in triplicate and measured in triplicate.

### Online Multimedia Supplements

The online data supplements (Supplementary Videos 1–4) contain movie files demonstrating migration assays of cPMNs and oPMNs performed as described in chemotaxis assays.

### Statistics

Chemotactic index, velocity, and speed of chemotactically active PMNs are presented in Tukey box and whisker plots (designed with GraphPad Prism, version 6.07, La Jolla, CA, USA). Chemotaxis data distribution was assessed with a D'Agostino- Pearson omnibus test and found to be not normally distributed. Statistical analyses of these data were performed by Friedman tests using GraphPad Prism software. Phagocytosis (adhesion, internalization, and killing assays), fMLP receptor expression, and NET formation data were normally distributed according to D'Agostino-Pearson omnibus tests. These data were analyzed with paired *t*-tests using GraphPad Prism software and presented as means + standard error of means (SEM). *P* < 0.05 were considered significant.

## Results

### oPMNs Exhibit Impaired Directional Chemotactic Accuracy Toward fMLP

**Figure 1:**
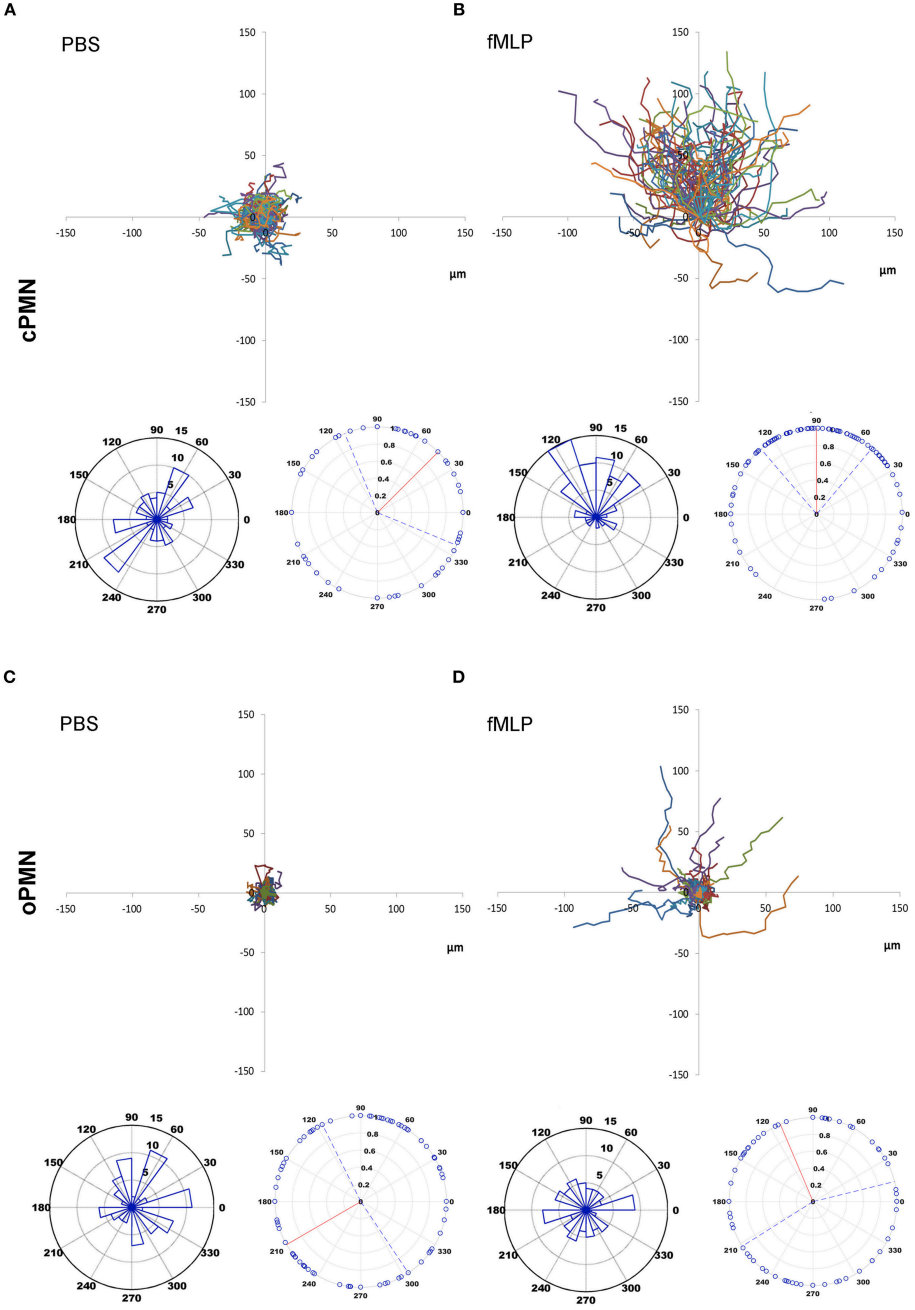
Chemotaxis of cPMNs and oPMNs. Spider, vector, and rose plots of cPMNs **(A,B)** and oPMNs **(C,D)** in response to PBS (left panels) and the chemoattractant fMLP (right panels) over a time period of 20 min. Each data set comprises three graphs: the top image is a spider plot of individual cell movement tracks. Under each spider diagram, two circular diagrams are presented; illustrating the direction of migrating cells over the whole time period of 20 min. The original (in the actual experimental setup) position of PBS **(A,C)** and the chemotactic agent fMLP **(B,D)** is at the North position on all graphs. Spider diagrams show the distance and direction of movement from the original position. Each individual line illustrates the complete movement (μmeter) of one PMN over the whole time period of 20 min. Vector plots (left) demonstrate the proportion of cells (5, 10 or 15 cells per chart) in segments moving toward any given direction from their original position. Rose plots (right) demonstrate the distribution of the final angle of all cells with a vector line showing the median angle (red vector) including the interquartile range (interrupting blue vectors). All diagrams are represented on the same scale. *n* = 9.

The chemotactic movements of cPMNs (Figures 1A,B) and oPMNs (Figures 1C,D) toward PBS and fMLP over the entire time period of 20 min were compared (See Supplementary Videos). For all datasets, an equal number of PMNs were tracked, analyzed and presented in spider, vector and rose plots (Figure 1).

Control conditions (PBS) of cPMNs (Figure 1A) and oPMNs (Figure 1C), as expected, show very little movement without any obvious directionality of movement. The strength of the movement, evidenced by the length of cell tracks in spider plots and the distribution of dots in rose plots (Figures 1A,C), is evenly distributed indicating non-directional, minimal random movement of cells in response to PBS. cPMNs show obvious chemotactic responses to fMLP compared to control treated cells, as previously described by Roberts et al. (16) (Figure 1B). cPMNs showed a strong response evidenced by longer cell tracks (spider diagrams), with the largest proportion of cells moving North (primarily 120°, vector plot), showing a “Northern Hemisphere” distribution of cells (rose plot) and a median strength of 90° (rose plot) toward fMLP. When comparing fMLP conditions, cPMNs (Figure 1B) have longer cell tracks than oPMNs (Figure 1D), where only a few cells show random chemotactic movement. oPMNs show no collective directional movement toward fMLP (Figure 1D). Overall, oPMN conditions showed similar movement patterns in control and treated conditions indicating that oPMNs exhibit impaired directional chemotactic movement toward the chemoattractant fMLP.

### oPMNs Are Live, Active Cells With Impaired Directional Migration Capacities

**Figure 2:**
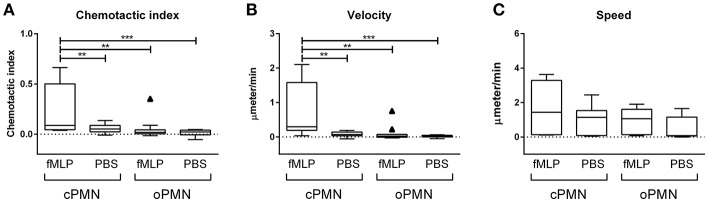
Chemotactic index, velocity, and speed of cPMNs and oPMNs in response to fMLP or PBS. Quantitative analysis of migration is presented in Tukey box and whiskers plots. The box represents the 75 and 25th percentiles with the median presented as a midline. Tukey whiskers represent the minimum and maximum values and outliers were defined as values exceeding 1.5 times the interquartile range. The chemotactic index, which is the directional accuracy of chemotaxis, is presented in **(A)**. The cell velocity, representing the average speed (μmeter/min) of the cells moving in its most prominent direction, is presented in **(B)**. The speed, which is the average speed (μmeter/ min) moving in any direction is presented in **(C)**. *n* = 9, ^**^p < 0.01, ^***^p < 0.001.

Quantitative analysis of migration by cPMNs and oPMNs in response to PBS and the chemotactic agent fMLP is shown in Figure 2. The chemotactic index (directional accuracy of chemotaxis) of cPMNs migrating toward fMLP is significantly higher than in all other conditions (Figure 2A). The same trend was observed for the average velocity of the cells in its most prominent direction over the time course (Figure 2B). In response to fMLP, a significantly lower velocity was observed in oPMNs when compared to cPMNs. No significant difference in chemotactic index and velocity was observed for oPMNs in response to PBS or fMLP (Figures 2A,B). The chemotactic index (Figure 2A), velocity (Figure 2B), and speed (Figure 2C), did not differ significantly between cPMN control conditions (PBS) and oPMNs in response to PBS or fMLP. The speed of cPMNs exposed to fMLP was slightly higher (1.53 ± 0.44 μm/min) but not significantly different than under control conditions (0.93 ± 0.26 μm/min) or of that observed for oPMNs (fMLP: 0.91 ± 0.23, PBS: 0.63 ± 0.21 μm/min). Altogether, with the exception of speed, oPMNs were not significantly different in their response to fMLP in comparison to control-treated cPMNs and oPMNs (Figure 2, compare Figures 1A,D). This data demonstrates that oPMNs are active, live cells with impaired migration capacities toward the chemotactic agent fMLP.

### Impaired Directional Migration Accuracy of oPMNs Toward fMLP Is Explained by Low fMLP Receptor Expression

**Figure 3:**
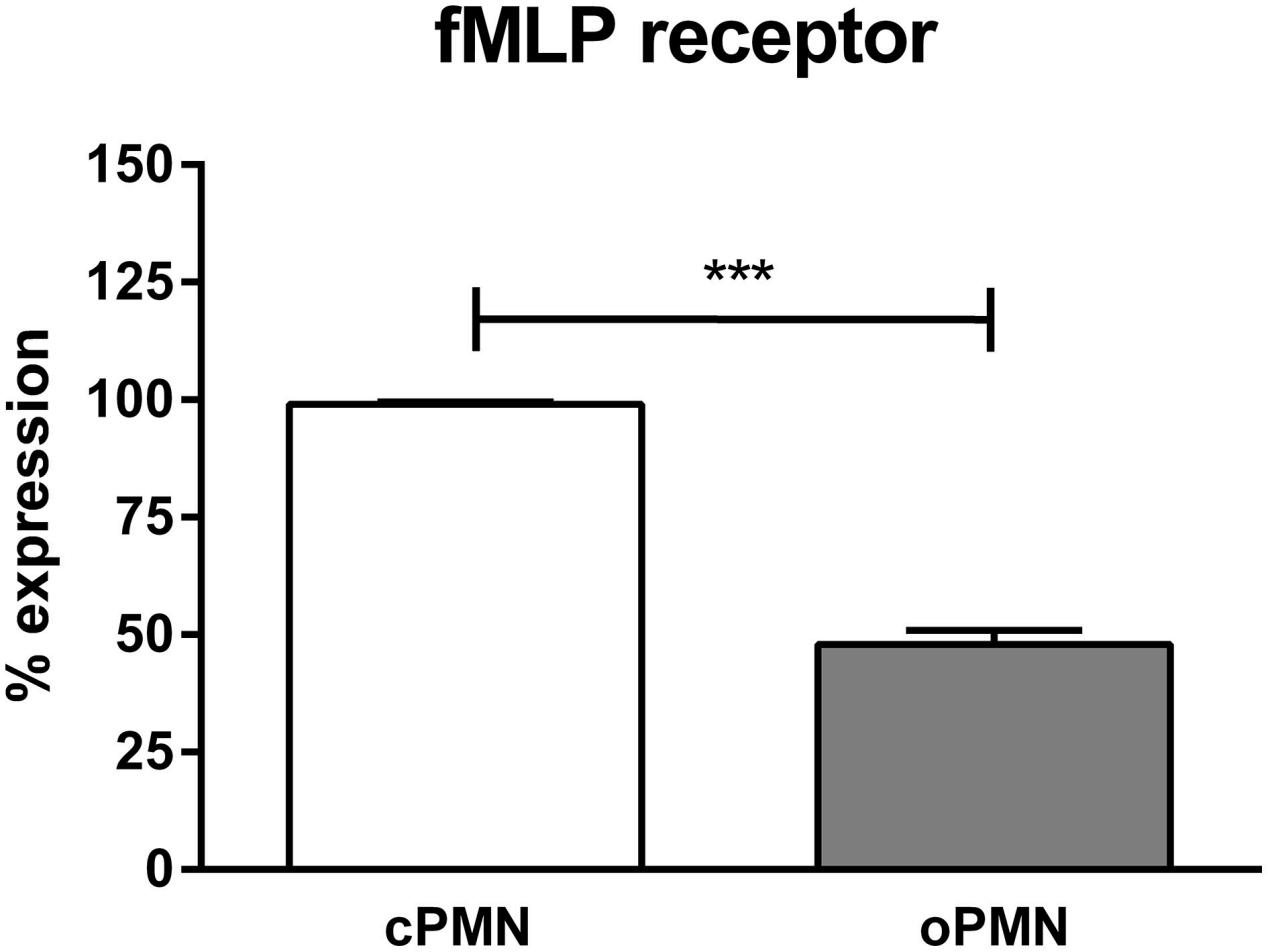
fMLP receptor expression by cPMNs and oPMNs. Quantitative analysis of fMLP receptor expression (percentages) of cPMNs (white bar) and oPMNs (gray bar). The gating strategy as presented in Supplementary Figure 2 was followed. Data are presented as mean (+SEM) percentages of fMLP receptor expression of the live, CD16+ population. *n* = 3, ^***^p < 0.0005.

Since oPMNs exhibited an impaired directional migration accuracy toward fMLP, we hypothesized that fMLP receptors on oPMNs were saturated and therefore incapable of binding further fMLP, rendering oPMNs incapable of migrating toward fMLP. As such, the expression of the fMLP receptor was analyzed on viable cPMNs and oPMNs using flow cytometry (Figure 3). Gating strategies employed for these experiments are shown in Supplementary Figure 2. Firstly, PMN surface markers CD66b and CD16 were tested on both cPMNs and oPMNs. Both cPMNs and oPMNs expressed CD16 (99.7 ± 0.2% and 96.2 ±1.2%, respectively, percentages ± SEM) and CD66b (99.8 ± 0.03% and 80.4 ± 6.5%, respectively, percentages ± SEM). Secondly, in these PMN populations, fMLP receptor expression was tested. Indeed, fewer oPMNs expressed the fMLP receptor (47.9 ± 3.0%), whereas nearly all cPMN were positive for the fMLP receptor (99.0 ± 0.6%, *p* < 0.0005).

### A Trend for Greater Adhesion by oPMNs than by cPMNs Was Observed

**Figure 4:**
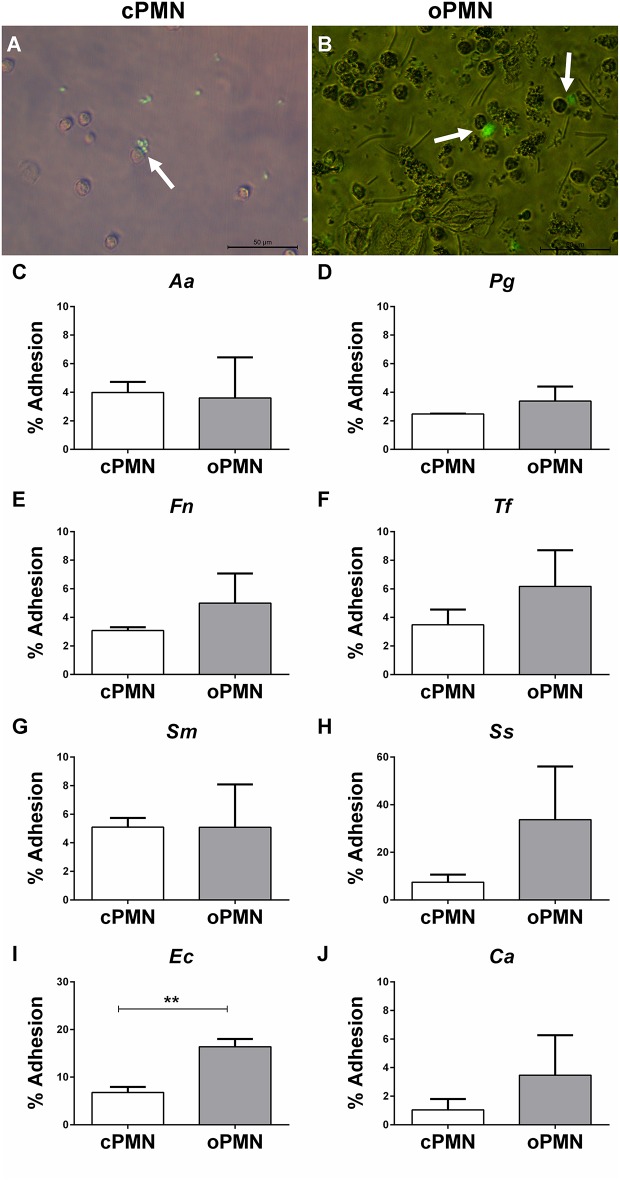
Adhesion by cPMNs and oPMNs. Micrographs of FITC labeled bacterial adhesion by cPMNs **(A)** and oPMNs **(B)**. Arrows depict adhesion of FITC labeled bacteria (*Fusobacterium nucleatum)* which are shown in green. All micrographs are representatives of 3 independent experiments. Scales represent 50 μm. Quantitative analysis of adhesion of heat-inactivated *A. actinomycetemcomitans* [*Aa*, **(C)**], *P. gingivalis* [(*Pg*, **(D)**], *F. nucleatum* [*Fn*, **(E)**], *T. forsythia* [*Tf*, **(F)**], *S. mutans* [*Sm*, **(G)**], *S. Sanguinis* [*Ss*, **(H)**], *E. coli* [*Ec*, **(I)**], and *C. albicans* [*Ca*, **(J)**] by cPMNs (white bars) and oPMNs (gray bars). The gating strategy presented in Supplementary Figure 3 was employed for all quantifications. Data are presented as mean (+SEM) percentages of adhesion, note the variation in y-axis scales. *n* = 3, ^**^ *p* < 0.01.

*In vivo*, PMNs migrate and adhere to pathogens in order to affect phagocytosis and the subsequent destruction of pathogens. Adhesion was investigated at 4°C in order to prevent active internalization. cPMN and oPMN adhesion were investigated with *Aa, Pg, Fn, Tf*, *Sm, Ss, Ec, and Ca*. Adhesion by cPMNs (Figure 4A) and oPMNs (Figure 4B) of FITC labeled microbes are illustrated by white arrows in Figure 4. Oral rinse samples contained oPMNs, epithelial cells, bacteria, and debris. Filtration with 10.0 μm filters excluded most of the epithelial cells, however, a limited amount of epithelial cells, bacteria, and small debris remained in these oPMN suspensions as seen in Figure 4B. Nevertheless, the purity of PMNs in these samples, as established by flow cytometry with CD16 and CD66b markers, was on average 99.7 and 99.8% for cPMNs, and 96.2 and 80.4% for oPMNs, respectively (Supplementary Figure 3). Adhesion of FITC-labeled micro-organisms by cPMNs and oPMNs, as shown in Figures 4A,B, was quantified using flow cytometry. The employed gating strategy is shown in Supplementary Figure 3. The graphs in Figures 4C–J show a trend of greater adhesion by oPMNs vs. cPMNs for the majority of the tested micro-organisms. In general, more oPMNs adhered to *Fn, Ca, Tf*, and *Ss*, although this difference did not reach statistical significance. The adhesive capacity of oPMNs to *Ec* and *Ss* was 2–3 times greater than for other tested microbes. Specifically, the adhesion of *Ec* was significantly higher (*p* = 0.0018) by oPMNs in comparison to cPMNs.

### oPMNs Exhibit an Increased Internalization Capacity of *Aa, Pg*, and *Ec*

**Figure 5:**
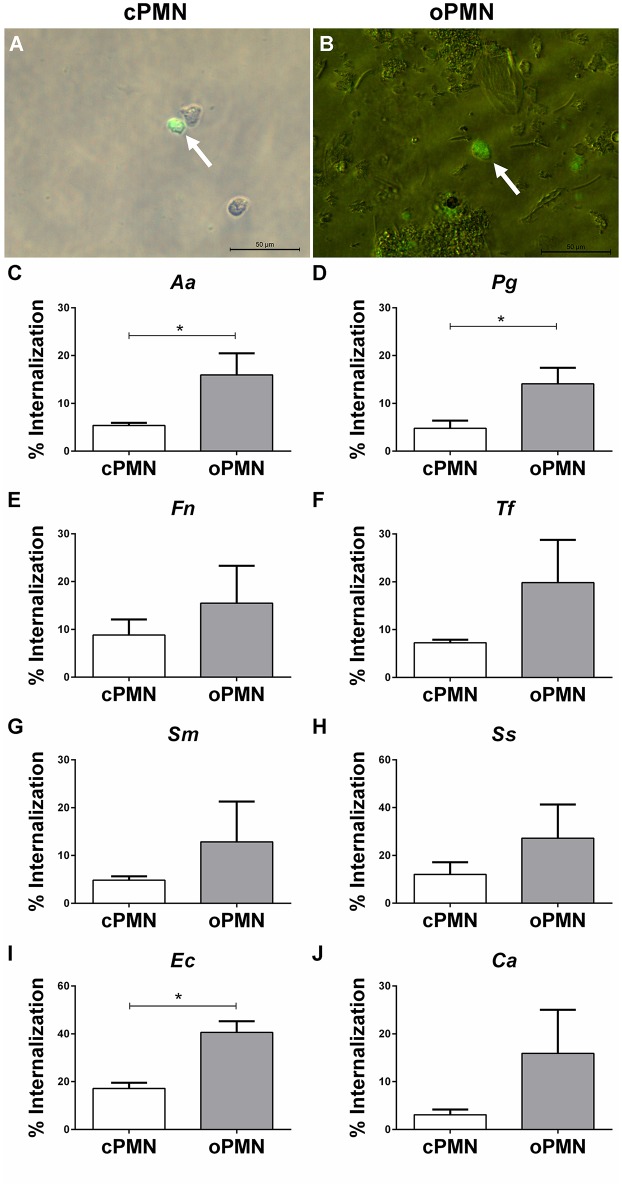
Internalization by cPMNs and oPMNs. Micrographs of FITC labeled internalization by cPMNs **(A)** and oPMNs **(B)**. White arrows depict adhesion of FITC labeled bacteria (*Fusobacterium nucleatum*) which are shown in green. All micrographs are representatives of 3 independent experiments. Scales represent 50 μM. Quantitative analysis of internalization of heat-inactivated *A. actinomycetemcomitans* **[***Aa*, **(C)**], *P. gingivalis* [*Pg*, **(D)**], *F. nucleatum* [*Fn*, **(E)**], *T. forsythia* [*Tf*, **(F)**], *S. mutans* [*Sm*, **(G)**], *S. Sanguinis* [*Ss*, **(H)**], *E. coli* [*Ec*, **(I)**], and *C. albicans* [*Ca*, **(J)**] by cPMNs (white bars) and oPMNs (gray bars). The gating strategy presented in Supplementary Figure 3 was employed for all quantifications. Data are presented as mean (+SEM) percentages of internalization, note the variation in y-axis scales. *n* = 3, ^*^p < 0.05.

Internalization by cPMNs (Figure 5A) and oPMNs (Figure 5B) of FITC labeled microbes is indicated with white arrows in Figure 5. Internalization of pathogens by cPMNs and oPMNs, as shown in Figures 5A,B, was quantified using flow cytometry. The gating strategy employed for flow cytometry experiments is shown in Supplementary Figure 3.

A clear trend for more internalization of all tested microbes by oPMNs than cPMNs is shown in graphs of Figures 5C–J. Similar to the adhesion assay results, *Ec* phagocytosis by oPMNs was significantly higher (*p* = 0.0042) than by cPMNs. Accordingly, the internalization capacity of oPMNs for *Ec* and *Ss* was approximately 2 times greater than for other tested microbes. The internalization of the periodontal bacteria *Aa* and *Pg* was 3-fold higher in oPMNs than in cPMNs. *Fn, Tf*, *Sm, Ss*, and *Ca* internalization by oPMNs did not differ significantly in comparison to cPMNs, however, there is a trend of more internalization by oPMNs. Interestingly, significantly more bacteria were internalized than adhered to by both cPMNs and oPMNs. In conclusion, oPMNs are viable, active cells with functional adhesion and internalization properties.

### oPMNs Have a Minimal Capacity to Kill *Ec*

The microbial killing capacity of cPMNs and oPMNs was assessed with live *Ec*. cPMNs were originated from a nearly sterile environment while oPMNs originate from an environment containing numerous oral bacteria. Therefore, oPMN samples were pre-incubated with antibiotics and subsequently plated in order to test oral bacteria survival. Incubation with antibiotics was sufficient to eliminate oral bacterial contamination in the oPMN samples as no more than 5 CFU were counted after overnight incubation of oPMN samples on BHI agar plates. No CFU were present on plated cPMN samples.

**Figure 6:**
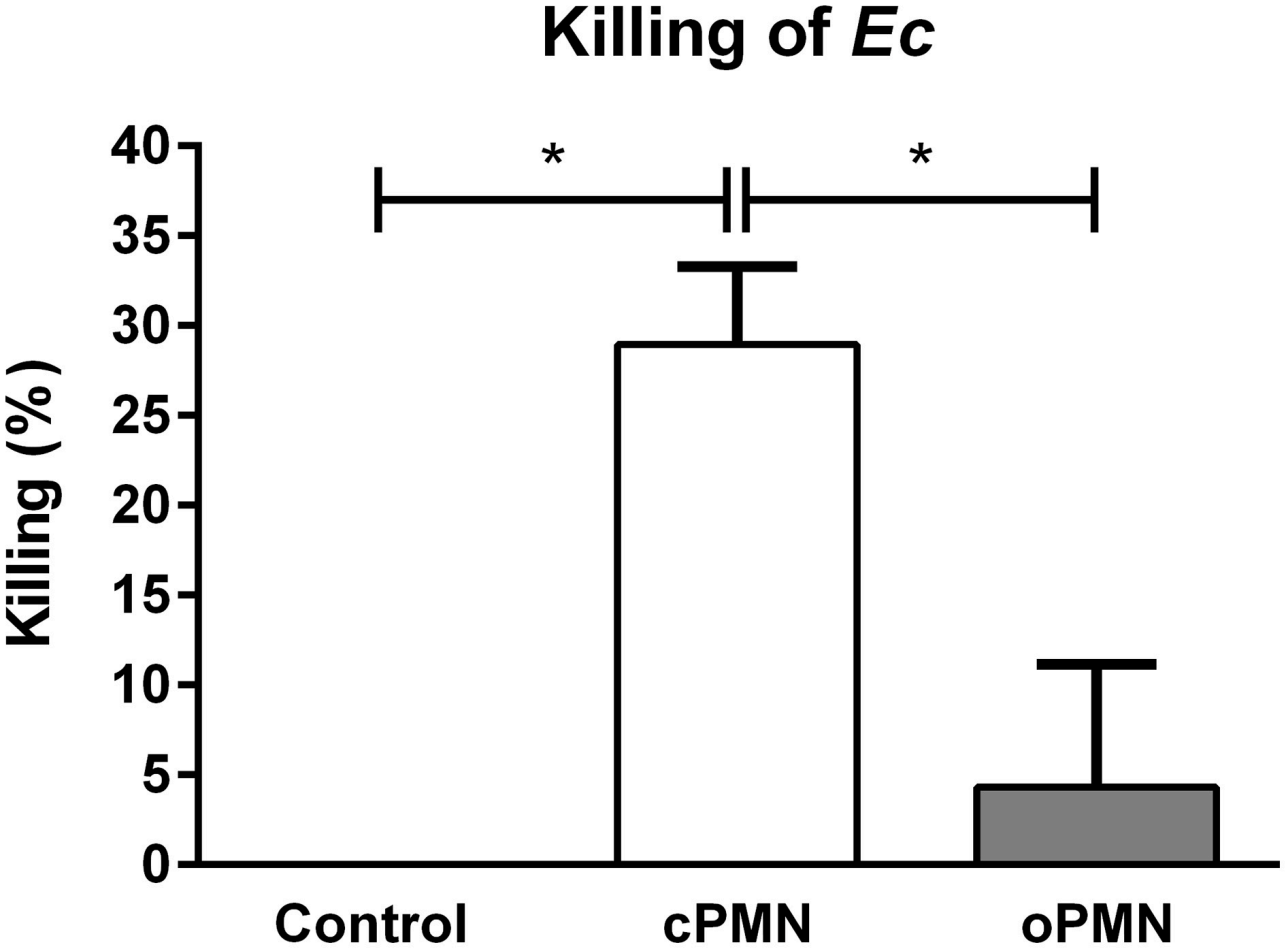
oPMN have a limited ability for intracellular killing of *Ec*. Quantitative analysis of killing capacity (percentages) of cPMNs (white bar) and oPMNs (gray bar). Percentages are based on the presence of CFU on overnight *Ec* agar plate assays. The control condition, representing 0% killing, was *Ec* incubated alone. On average, cPMNs killed 29 ± 4% while oPMNs killed 4 ± 7%. cPMNs killed significantly more *Ec* than both the control condition (*p* = 0.02) and oPMNs (*p* = 0.02). Data are presented as mean percentages (+SEM), *n* = 4, ^*^p < 0.05.

Compared to the control condition, cPMNs after incubation with *Ec* for 30 min, showed reduced numbers of CFU after the overnight cultures, corresponding to a 29% increase in intracellular killing (*p* = 0.02, Figure 6). In contrast, oPMNs showed 4% killing under the same conditions. This was significantly lower than cPMNs (*p* = 0.02) and the killing by oPMNs did not differ significantly from the control condition. We conclude that oPMNs show a minimal ability for the intracellular killing of *Ec*.

### Hyper(re)active NET Formation by Unstimulated oPMNs

**Figure 7:**
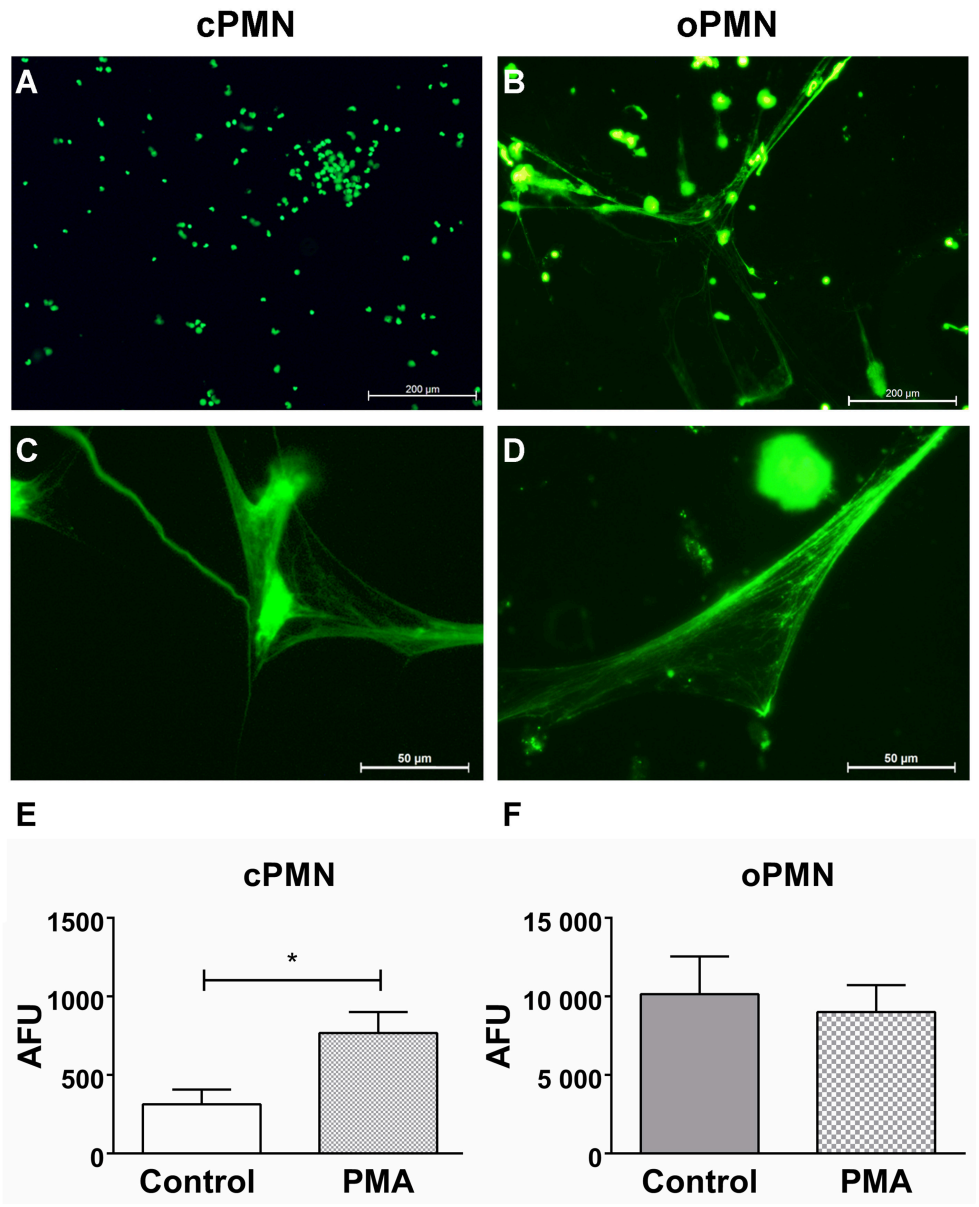
NET formation by unstimulated and stimulated cPMNs and oPMNs. Micrographs of cPMNs (left panels) and oPMNs (right panels) under unstimulated **(A,B)** and stimulated conditions **(C,D)**. Significant differences are observed for cPMNs between unstimulated (control, white bar) and stimulated (PMA, gray bar) conditions **(E)**. All micrographs are representatives of 9 independent experiments. No significant difference (*p* = 0.70) was observed between unstimulated (control, gray bar) and stimulated (pattern, PMA) conditions of oPMN NET formation **(F)**. Quantitative NET formation data are presented as mean (+SEM) arbitrary fluorescent units (AFU), note the variation in y-axis scales. *n* = 9, ^*^p < 0.05.

Previous research from our group reported a significant increase in extracellular ROS production by oPMNs when compared to cPMNs from healthy donors (7). Here, we investigated another antimicrobial property of PMNs: the formation of NETs. NET formation by cPMNs has been widely studied and confirmed *in vivo* and *ex vivo* using various functional assays. However, the ability of oPMNs to produce NETs was unknown. NET release by cPMNs and oPMNs was visualized (Figures 7A–D) and quantified (Figures 7E,F) using the membrane impermeable extracellular DNA dye SYTOX™ green. Immunofluorescence microscopy analyses showed an absence of NETs in unstimulated (culture medium) cPMNs (Figure 7A). After stimulation with a well-established NET-inducer PMA (39), both oPMNs and cPMNs were capable of producing NETs (Figures 7C,D). Fluorimetric quantification of NET formation demonstrated significantly more NETs in stimulated conditions (PMA) by cPMNs in comparison to unstimulated control conditions (Figure 7E). Interestingly, oPMNs showed apparent NET release in both unstimulated (control) and stimulated (PMA) conditions (Figure 7B), where no significant difference was observed between unstimulated and stimulated conditions for cPMNs (Figure 7F). SYTOX™ green is a non-membrane-permeable nucleic acid dye which also stains Gram positive and negative bacteria (40). Accordingly, Figure 7D shows that NETs produced by oPMNs are saturated with oral bacteria co-isolated from the oral cavity, which illustrates the major function of NETs: entrapping and immobilizing bacteria. Unstimulated oPMNs show 32-fold increased NET formation over unstimulated cPMNs and 13-fold increased NET formation when compared to stimulated cPMNs, demonstrating the hyperactive phenotype of oPMNs.

## Discussion

The predominant immune cell that is constitutively recruited into the oral cavity is the PMN, being a protective and antimicrobial innate immune responder. In healthy conditions, about 30,000 PMNs transit per minute from the circulatory blood into the oral mucosal tissues and gingival crevices. The crucial role of PMNs in (oral) health maintenance has been extensively studied. However, the majority of studies have focused on cPMNs, which may not necessarily reflect the roles and characteristics of PMNs within the oral cavity. Hitherto, little is known about the functional role of orally derived PMNs in relation to oral health and inflammation. Therefore, we aimed to compare oPMNs and cPMNs, in terms of their chemotactic, phagocytic, and NET forming capacities. The main findings of the current study are that oPMNs obtained from oral rinses are viable, active cells with impaired directional chemotactic accuracy toward fMLP and a pattern of low fMLP receptor expression, rendering them terminally migrated cells. oPMNs adequately perform innate immune responses including adhesion and internalization of various microbes, NET formation, and, as previously investigated by our group, extracellular ROS production. Interestingly, adhesion and internalization by oPMNs of *Ec* was accompanied by a minimal capacity for intracellular killing. Collectively, the results demonstrate that oPMNs have functional potential. With increased adhesion, internalization and NET formation, oPMNs most likely contribute to maintaining a balanced oral ecosystem.

PMNs are short-living cells with an estimated half-life of 6–8 h that remain in the circulation for a few hours before they extravasate into tissues (41). Obviously, oPMNs are more mature cells than cPMNs due to their transendothelial extravasation, oral transepithelial migration and exposure to the oral biofilm. Studies show that oPMNs, which have migrated from the circulatory blood into the oral cavity, have undergone functional changes characterized by the increased production of pro-inflammatory cytokines and enhanced apoptosis mechanisms (7, 12). Once within the oral cavity, PMNs are exposed to a lower osmolality in saliva than in blood—rapid cell swelling and eventually lysis of PMNs in response to those osmolities found in saliva have been reported (42). In our setup, all experiments were completed within 3 h after isolation, and cells were tested for viability. oPMNs were washed and stored in a physiological isotonic buffer (same osmolality as blood), minimizing the inhibitory effect of hypotonic saliva. Furthermore, cPMNs incubated with saliva do not exhibit functional responses similar to oPMNs, indicating that the oPMNs' phenotype is altered due to local characteristics such as transmigration and interactions with the oral biofilm and not by the mere exposure to saliva (43).

*In vivo*, PMNs migrate from the vasculature into the oral cavity in order to affect phagocytosis and the subsequent destruction of pathogens by degranulation, ROS, and NET production. Effective PMN recruitment, invasion, and activation are crucial antimicrobial functions for periodontal health maintenance. In the presence of chronic gingival inflammation, as apparent in periodontitis, oPMNs migrate into the oral cavity at an increased rate (33, 44). Hyperactive, hyperreactive, supernumerary, or dysregulated PMNs have been reported as key players in chronic inflammation as they can cause collateral tissue damage through the release of inflammatory and toxic substances or tissue-degrading enzymes (8, 45). Therefore, the functional properties of oPMNs were investigated. Impaired directional migration toward fMLP by cPMNs from periodontitis patients in comparison to cPMNs from healthy subjects has been reported by Roberts et al. (16). Here, we investigated oPMN migration toward the chemotactic agent fMLP and showed the dysfunctional movement of viable oPMNs, which was explained by low fMLP receptor expression.

PMNs are recruited from the vasculature into the gingival crevice and adhere to the epithelium which is regulated by intercellular adhesion molecule-1 (ICAM-I), expressed by mucosal keratinocytes (46) and gingival fibroblasts (47). Adhesion and migration across epithelial barriers are facilitated by increased CD11b expression in PMNs (48). Rijkschroeff et al. reported significantly higher CD11b expression by oPMNs when compared to cPMNs from healthy individuals. The expression, and thus migration facilitation of oPMNs, is thus partly managed by CD11b expression and likely exhausted after reaching the oral cavity. Impaired chemotactic accuracy by oPMNs could be explained by their journey through the oral mucosal tissues and exposure to the abundant oral biofilms. PMNs constantly interact with symbiotic and dysbiotic oral biofilms to maintain oral homeostasis and health (49). Exposure to numerous oral bacteria, as apparent in oPMN samples, would explain the low expression of fMLP receptors on oPMNs. Accordingly, a number of the oPMNs' fMLP receptors were likely saturated and therefore incapable of binding further fMLP, rendering PMNs unable to migrate toward fMLP. Almost all viable cPMNs expressed fMLP, explaining the higher sensitivity to fMLP as evident in our migration studies. Thus, oPMNs may be desensitized to fMLP, as was used in our migration assay. In this study, fMLP was chosen as a chemoattractant since it was previously reported as the most effective agent in migration assays, as evidenced by a significant CI, velocity, and speed of cPMNs from healthy donors (16). Here, no significant difference was observed between the speed of oPMNs and cPMNs indicating that oPMNs are viable cells.

Apart from impaired directional migration, decreased cPMN phagocytosis has been demonstrated to play a major role in the etiopathogenesis of periodontitis (50), and thus represents a very relevant function to study in oPMNs. Since phagocytosis is considered to be a multiple-step process, we investigated the adhesion and internalization of various heat-inactivated micro-organisms by cPMNs and oPMNs separately. Interestingly, oPMNs have increased phagocytic capacities when compared to cPMNs indicating that oPMNs are effective innate responders with a high potential for phagocytosis. Here, different species were used and showed distinct phagocytic responses by both cPMNs and oPMNs. Overall, the same trends were visible, however, not all microbes were adhered to or internalized similarly, demonstrating the importance of the use of different microbes in phagocytosis assays. Furthermore, significantly more bacteria were internalized than adhered to by both cPMNs and oPMNs. Although oPMNs showed an increased capacity to adhere to and internalize *Ec*, they were incapable of killing live *Ec*. It is possible that oPMNs remained capable of ingesting microbes while their actual digestion and destruction capacities were exhausted. We previously demonstrated elevated extracellular ROS production by oPMNs, which is a subsequent step to phagocytosis, further along the activation cascade of the PMN (44). Together with our new findings, showing hyperactive NET formation, we hypothesize that the oPMNs' hyperactive state represents their an ultimate attempt to limit bacterial dissemination. Possibly, as oPMNs are incapable of degrading ingested microbes due to inefficient phagolysosome formation and granule perishment, they proceed to enter their final state: NETosis (51). Nevertheless, as previously shown by others (52, 53), some bacteria are capable of escaping NETs.

Lastly, the NET formation capacities of cPMNs and oPMNs were investigated. We demonstrated that oPMNs produce 13 times more NETs than stimulated cPMNs in both unstimulated and stimulated conditions. Thus, after arriving in the oral cavity, oPMNs are in a hyperreactive activation state as evidenced by increased NET and ROS formation activity (44). Unstimulated NET formation capacities could be explained by the previously reported hyperreactive activation state of oPMNs (12, 54, 55) and by the extracellular environment of oPMNs. In contrast to cPMNs, the extracellular environment of oPMNs is contaminated with saliva, oral bacteria, epithelial cells, and cell debris. Since NETs are reportedly induced by bacterial products (24, 56), the hyperactive NET production of oPMNs is likely influenced by the contaminated extracellular environment. Accordingly, oPMNs are constantly stimulated by this extracellular environment containing oral biofilm components and salivary microorganisms, which may explain the significantly increased rates of NET formation, even in unstimulated conditions.

Undoubtedly, a non-vital PMN is unable to migrate or move in a minimal, non-directional fashion, and adhere to or internalize microbes, as shown in our experiments. Extracellular conditions and the activation state of a PMN can influence whether PMNs undergo NETosis or phagocytose a pathogen (51). Ineffective phagocytosis will possibly prompt the production of ROS and NETs. About 20–30% of cPMNs produce NETs *in vitro* (22, 23). It is unlikely that after NETosis, a PMN could subsequently perform its live, antimicrobial functions and then die by apoptosis. Accordingly, evidence exists for distinct phenotypic subsets of neutrophils based on the expression of cell surface markers (12, 57–59), suggesting large phenotypic heterogeneity and functional versatility. In this study, different subsets were not investigated, since we were interested in the whole PMN population present in the oral cavity and in comparing this population to circulatory PMNs. However, it is conceivable that some oPMNs solely perform phagocytosis, while others are more committed to produce (suicidal) NETs.

In conclusion, the terminally migrated oPMN is a viable cell with a hyperactive phenotype, as evidenced by increased adhesion and internalization of microbes, and NET formation capacities. These findings contribute to a better understanding of the role PMNs play in maintaining oral health.

## Ethics Statement

The study was approved by the Birmingham ethical committee (14/SW/1148) and the Medical Ethical Committee of the Amsterdam University Medical Center, The Netherlands (2012-210#B2012406). All donors were systemically and periodontally healthy. Informed and written consent was obtained from all individuals prior to inclusion. Venous blood samples and oral rinses from each donor were collected.

## Author Contributions

CM, BL, and EN contributed to the conception and design of this study. CM performed all experiments, analyses, data interpretations, and wrote the first draft of the manuscript. JH helped with chemotaxis, killing, and NET formation experiments. LC assisted with adhesion and internalization experiments. EN and BL supervised the study. CM, JH, LC, IC, BL, and EN contributed to manuscript revisions, read, and approved the submitted version.

### Conflict of Interest Statement

The authors declare that the research was conducted in the absence of any commercial or financial relationships that could be construed as a potential conflict of interest.

## Supplementary Material

Supplementary Figure 1 Viability of cPMNs and oPMNs. Micrographs of cPMNs **(A)** and oPMNs **(B)** stained with the membrane-impermeable dye propidium iodide. Accordingly, dead cells are shown in red (indicated with an arrow). Note the contamination of epithelial cells and debris in the oPMN population **(B)**. Images were captured at a 10x magnification, scale bars represent 200 μm. The viability of cPMNs [95.30%, **(C**,**D)**] and oPMNs [70.12%, **(E,F)**] is shown and was determined with the Muse™ count & viability kit, which makes use of a membrane-permeant DNA staining dye to distinguish between (nucleated) live and dead cells. Viability is shown on the x-axis for all plots. Cell size index is shown on the y-axes of **(C,E)**, which distinguishes the cells (green area) from debris (gray area). Nucleated cells are displayed on the y-axes of **(D,F)**, discriminating the live nucleated cells (green area) from the non-nucleated dead cells and debris (gray area).

Supplementary Figure 2 Gating strategy for fMLP receptor expression experiments. Gating strategy is presented for cPMNs (left panels) and oPMNs (right panels). The live population was determined based on forward (FSC) and sideward scatter (SSC), representing, respectively the distribution of cells in the light scatter based on size and intracellular composition [**(A,B)**, encircled in red]. Furthermore, CD66b [CD66b+ populations, **(D,F)]** and CD16 [CD16+ populations, **(H,J)]** expression by the live population was assessed for cPMNs and oPMNs, respectively. Unstained controls were used to determine CD66b negative [red gates, **(C,E)**] and CD16 negative gatings **(G,I)**. Finally, in the CD16+ population, fMLP receptor expression was assessed on cPMNs **(L)** and oPMNs **(N)**. fMLP receptor negative gates were set in control conditions, stained with the isotype IgG1κ **(K,M)**.

Supplementary Figure 3 Gating strategy for adhesion and internalization experiments. Gating strategies for cPMNs (left panels) and oPMNs (right panels) are shown. The live population was determined based on forward (FSC) and sideward scatter (SSC) [**(A,B)**, encircled in red]. CD66b expression (CD66b+ populations) by the live population was assessed with anti-CD66b staining for both cPMNs **(D)** and oPMNs **(F)**. Unstained controls were used to determine CD66b negative gates **(C,E)**. CD16 expression by the live PMN population was assessed for both cPMNs [FITC–CD16+ gate, **(G)**] and oPMNs [FITC–CD16+ gate, **(I)**]. Adhesion and internalization of FITC-labeled bacteria by PMNs was determined based on the percentage of FITC+CD16+ [green gates, **(H,J)**] population. Accordingly, this population illustrates the percentage of FITC+ events (i.e., FITC-labeled microbes) detected in the CD16+ (i.e., PMN) population.

Supplementary Video 1 cPMNs migrating toward PBS. cPMNs in response to PBS over a time period of 20 min. The original (in the actual experimental setup) position of PBS is at the North position. Each individual line illustrates the (complete) movement of one cPMN over the whole time period of 20 min. In total, 15 random cells were tracked per condition. Scale bar represents 100 μm. This video is a representative of 9 independent experiments.

Supplementary Video 2 cPMNs migrating toward fMLP. cPMNs in response to the chemoattractant fMLP over a time period of 20 min. The original (in the actual experimental setup) position of the chemotactic agent fMLP is at the North position. Each individual line illustrates the (complete) movement of one cPMN over the whole time period of 20 min. In total, 15 random cells were tracked per condition. Scale bar represents 100 μm. This video is a representative of 9 independent experiments.

Supplementary Video 3 oPMNs migrating toward PBS. oPMNs in response to PBS over a time period of 20 min. The original (in the actual experimental setup) position of PBS is at the North position. Each individual line illustrates the (complete) movement of one oPMN over the whole time period of 20 min. In total, 15 random cells were tracked per condition. Scale bar represents 100 μm. This video is a representative of 9 independent experiments.

Supplementary Video 4 oPMNs migrating toward fMLP. oPMNs in response to the chemoattractant fMLP over a time period of 20 min. The original (in the actual experimental setup) position of the chemotactic agent fMLP is at the North position. Each individual line illustrates the (complete) movement of one oPMN over the whole time period of 20 min. In total, 15 random cells were tracked per condition. Scale bar represents 100 μm. This video is a representative of 9 independent experiments.

## Abbreviations

μm: micrometer
*Aa*: *Aggregatibacter actinomycetemcomitans*
BHI: brain-heart infusion
*Ca*: *Candida albicans*
CD: cluster of differentiation
CI: chemotactic index
cPMN: circulatory PMN
*Ec*: *Escherichia coli*
FITC: fluorescein isothiocyanate
fMLP: N-formyl-L-methionyl-L-leucyl-phenylalanine
*Fn*: *Fusobacterium nucleatum*
GCF: gingival crevicular fluid
ICAM: intercellular adhesion molecule-1
IL: interleukin
LPS: lipopolysaccharide
oPMN: oral PMN
*Pg*: *Porphyromonas gingivalis*
PI: propidium iodide
PMA: phorbol 12-myristate-13-acetate
PMN: polymorphonuclear leukocyte
ROS: reactive oxygen species
SEM: standard error of means
*Sm*: *Streptococcus mutans*
*Ss*: *Streptococcus sanguinis*
*Tf*: *Tannerella forsythia;*
TNF-α: tumor necrosis factor alpha.
