# Supplementary figures and images for: Oral Neutrophils Characterized: Chemotactic, Phagocytic, and Neutrophil Extracellular Trap (NET) Formation Properties
Source: Front Immunol. 2019 Mar 29;10:635. doi: 10.3389/fimmu.2019.00635 (PMC6449731; doi:10.3389/fimmu.2019.00635)

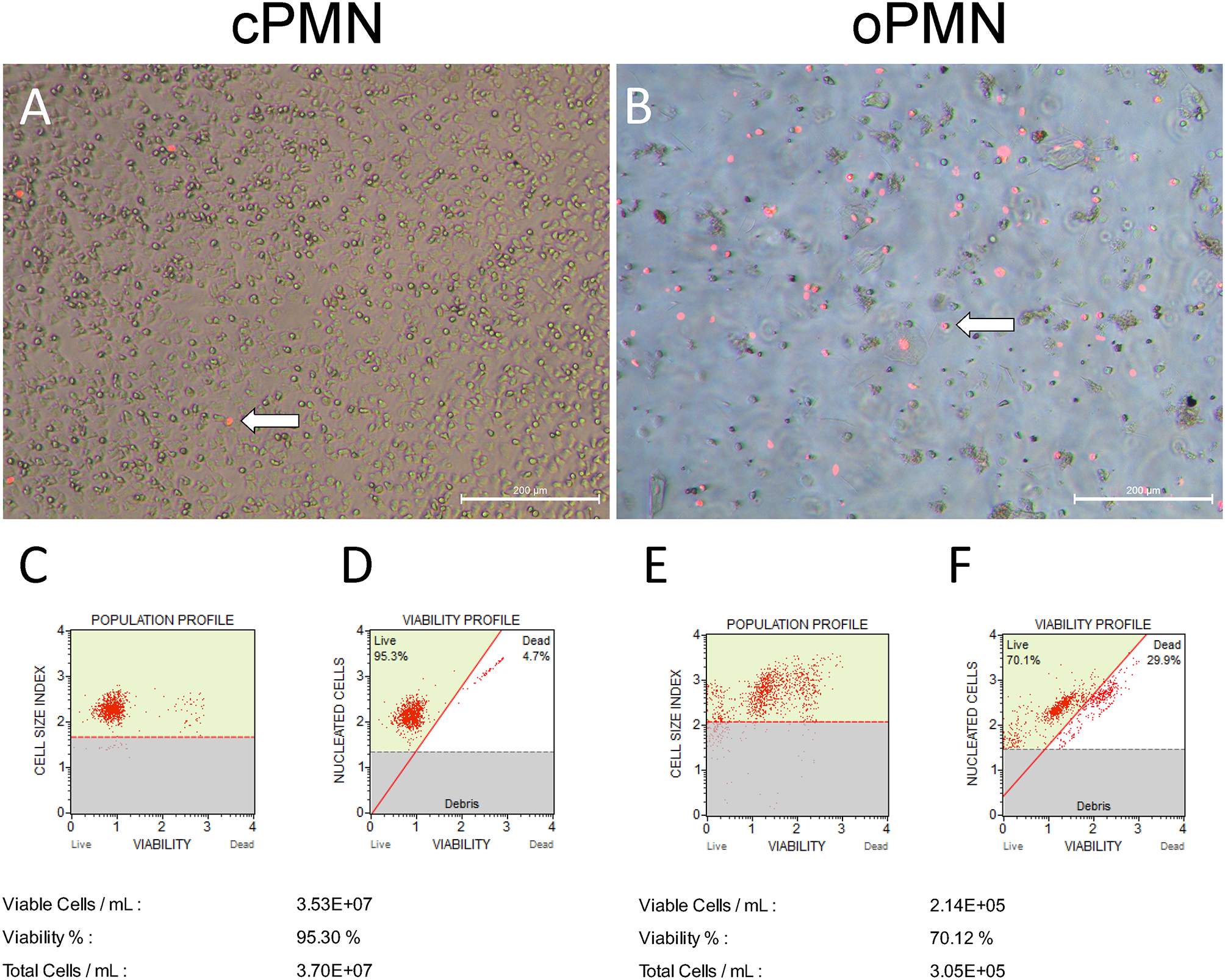

Supplement: Supplementary Figure 1 — Viability of cPMNs and oPMNs. Micrographs of cPMNs (A) and oPMNs (B) stained with the membrane-impermeable dye propidium iodide. Accordingly, dead cells are shown in red (indicated with an arrow). Note the contamination of epithelial cells and debris in the oPMN population (B). Images were captured at a 10x magnification, scale bars represent 200 μm. The viability of cPMNs [95.30%, (C,D)] and oPMNs [70.12%, (E,F)] is shown and was determined with the Muse™ count & viability kit, which makes use of a membrane-permeant DNA staining dye to distinguish between (nucleated) live and dead cells. Viability is shown on the x-axis for all plots. Cell size index is shown on the y-axes of (C,E), which distinguishes the cells (green area) from debris (gray area). Nucleated cells are displayed on the y-axes of (D,F), discriminating the live nucleated cells (green area) from the non-nucleated dead cells and debris (gray area). [file Image_1.TIF]

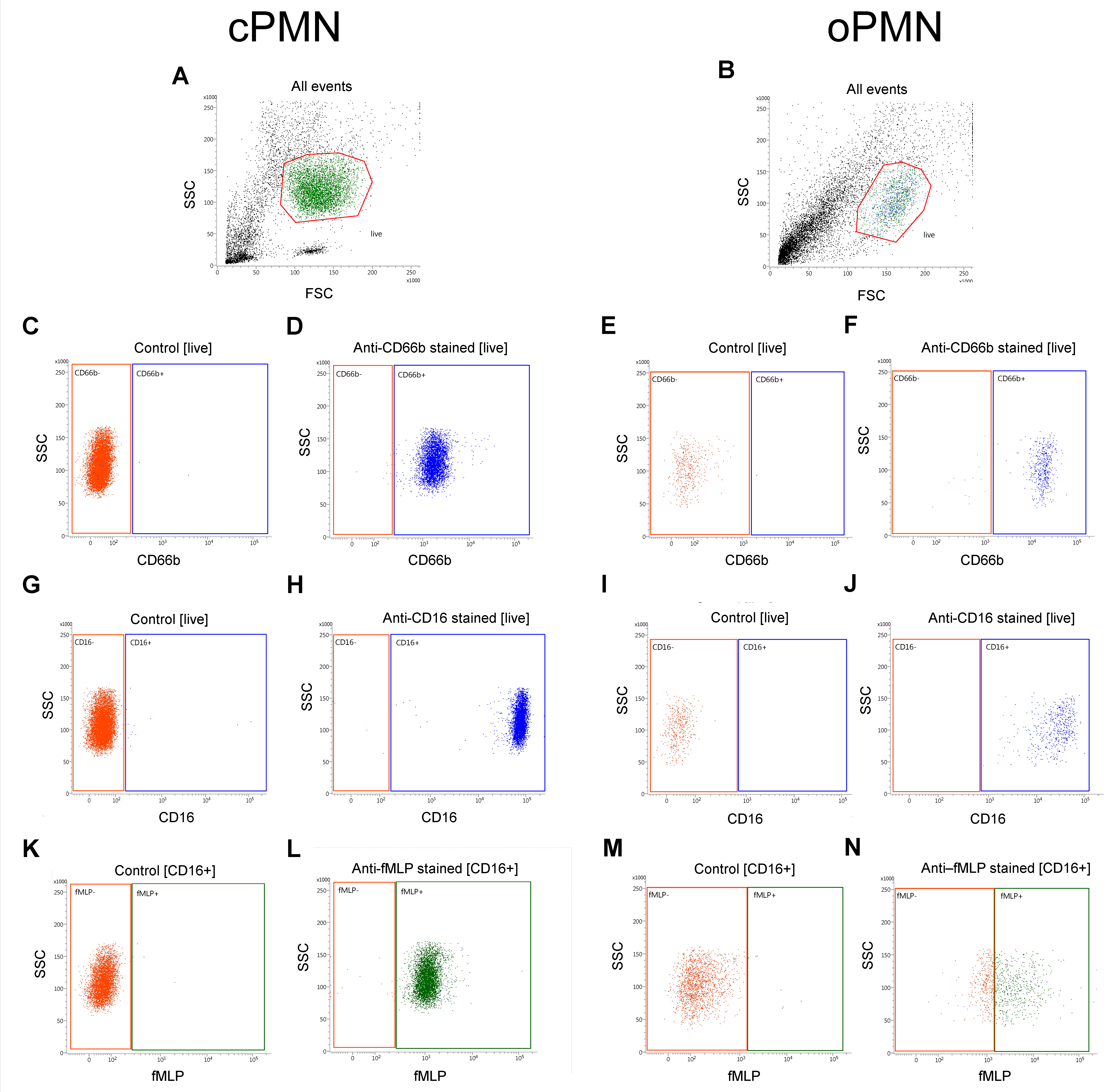

Supplement: Supplementary Figure 2 — Gating strategy for fMLP receptor expression experiments. Gating strategy is presented for cPMNs (left panels) and oPMNs (right panels). The live population was determined based on forward (FSC) and sideward scatter (SSC), representing, respectively the distribution of cells in the light scatter based on size and intracellular composition [(A,B), encircled in red]. Furthermore, CD66b [CD66b+ populations, (D,F)] and CD16 [CD16+ populations, (H,J)] expression by the live population was assessed for cPMNs and oPMNs, respectively. Unstained controls were used to determine CD66b negative [red gates, (C,E)] and CD16 negative gatings (G,I). Finally, in the CD16+ population, fMLP receptor expression was assessed on cPMNs (L) and oPMNs (N). fMLP receptor negative gates were set in control conditions, stained with the isotype IgG1κ (K,M). [file Image_2.TIF]

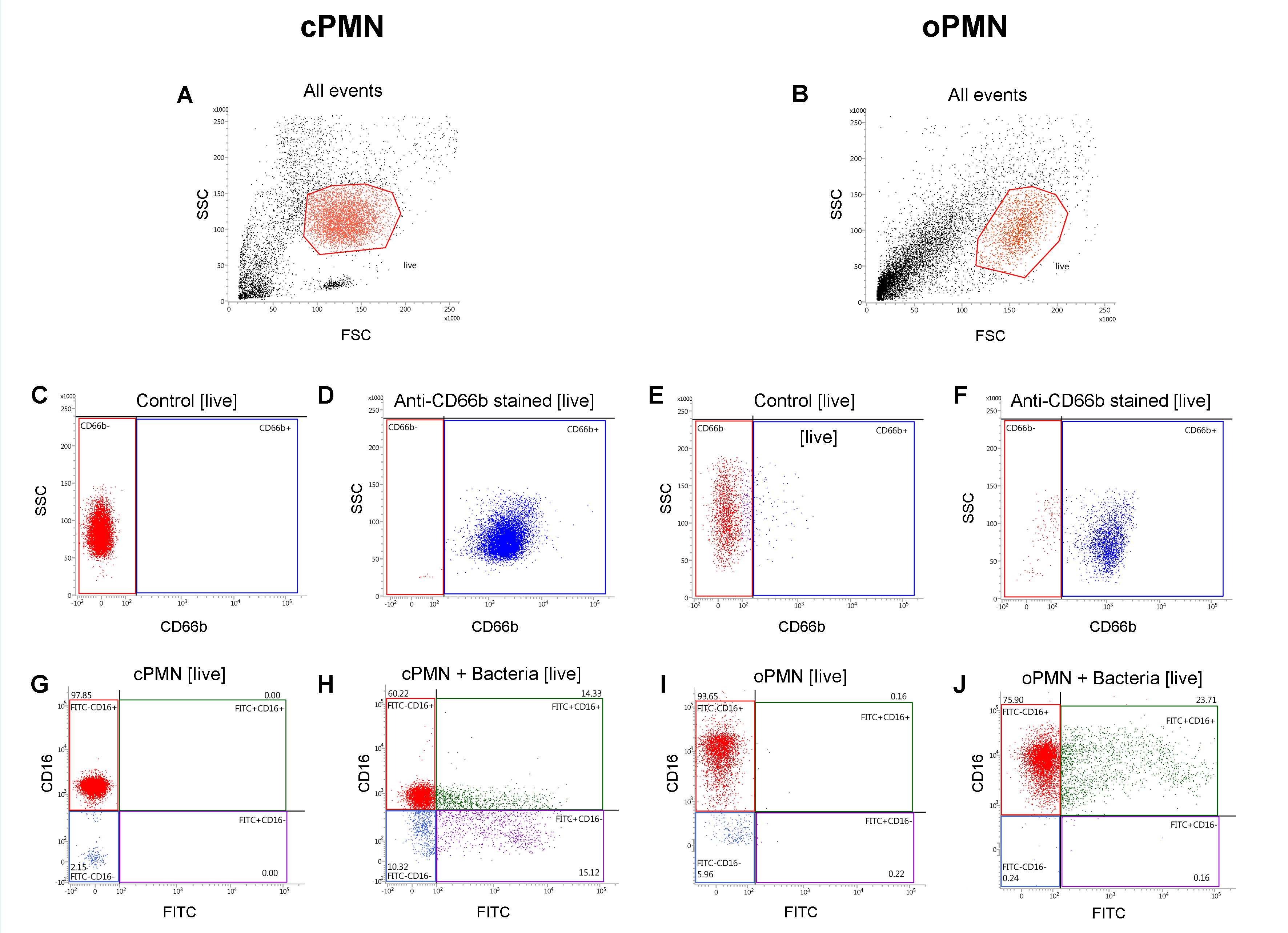

Supplement: Supplementary Figure 3 — Gating strategy for adhesion and internalization experiments. Gating strategies for cPMNs (left panels) and oPMNs (right panels) are shown. The live population was determined based on forward (FSC) and sideward scatter (SSC) [(A,B), encircled in red]. CD66b expression (CD66b+ populations) by the live population was assessed with anti-CD66b staining for both cPMNs (D) and oPMNs (F). Unstained controls were used to determine CD66b negative gates (C,E). CD16 expression by the live PMN population was assessed for both cPMNs [FITC–CD16+ gate, (G)] and oPMNs [FITC–CD16+ gate, (I)]. Adhesion and internalization of FITC-labeled bacteria by PMNs was determined based on the percentage of FITC+CD16+ [green gates, (H,J)] population. Accordingly, this population illustrates the percentage of FITC+ events (i.e., FITC-labeled microbes) detected in the CD16+ (i.e., PMN) population. [file Image_3.TIF]
